# Clients in Uganda accessing preferred differentiated antiretroviral therapy models achieve higher viral suppression and are less likely to miss appointments: a cross‐sectional analysis

**DOI:** 10.1002/jia2.26122

**Published:** 2023-07-06

**Authors:** Esther K. Karamagi Nkolo, Jessica Clinkscales Ejike, Simon Sensalire, Juliana Nabwire Ssali, Immaculate Ddumba, Jacqueline Calnan, Carolina Gonzalez, Nelly Maina, Melaku Dessie, Lauren Bailey, Ugochukwu Amanyeiwe, Thomas Minior, Karishma Srikanth, Herbert Kadama, Khushi Patel, Dina Patel

**Affiliations:** ^1^ U.S. Agency for International Development (USAID) Kampala Uganda; ^2^ GHTASC, Credence Management Solutions LLC, supporting the United States Agency for International Development (USAID), Office of HIV/AIDS Washington DC United States; ^3^ University Research Co., LLC USAID RHITES N‐Acholi Activity Kampala Kampala Uganda; ^4^ U.S. Agency for International Development (USAID), Office of HIV/AIDS, Prevention Care and Treatment Division Washington DC USA; ^5^ Ministry of Health AIDS Control Program Kampala Uganda; ^6^ Brown University [USAID Intern] Providence Rhode Island USA

**Keywords:** ART, client preference, DART, missed appointment, Uganda, viral load suppression

## Abstract

**Introduction:**

The Uganda Ministry of Health recommends facility‐ and community‐based differentiated antiretroviral therapy (DART) models to support person‐centred care for eligible clients receiving antiretroviral therapy (ART). Healthcare workers assess client eligibility for one of six DART models upon initial enrolment; however, client circumstances evolve, and their preferences are not routinely adjusted. We developed a tool to understand the proportion of clients accessing preferred DART models and compared the outcomes of clients accessing preferred DART models to the outcomes of clients not receiving preferred DART models.

**Methods:**

We conducted a cross‐sectional study. A sample of 6376 clients was selected from 113 referrals, general hospitals and health centres purposely selected from 74 districts. Clients receiving ART accessing care from the sampled sites were eligible for inclusion. Healthcare workers interviewed clients (caretakers of clients under 18), over a 2‐week period between January and February 2022 using a client preference tool to elicit whether clients were receiving DART services through their preferred model. Treatment outcomes of viral load test, viral load suppression and missed appointment date were extracted from clients’ medical files before or immediately after the interview and de‐identified. The descriptive analysis determined the interaction between client preferences and predefined treatment outcomes by comparing outcomes of clients whose care aligned with their preferences to outcomes of clients whose care misaligned with their preferences.

**Results:**

Of 25% (1573/6376) of clients not accessing their preferred DART model, 56% were on facility‐based individual management and 35% preferred fast‐track drug refills model. Viral load coverage was 87% for clients accessing preferred DART models compared to 68% among clients not accessing their preferred model. Viral load suppression was higher among clients who accessed the preferred DART model (85%) compared to (68%) clients who did not access their preferred DART model. Missed appointments were lower at 29% for clients who accessed preferred DART models compared to 40% among clients not enrolled in the DART model of their choice.

**Conclusions:**

Clients who accessed their preferred DART model have better clinical outcomes. Preferences should be integrated throughout health systems, improvement interventions, policies and research efforts to ensure client‐centred care and client autonomy.

## 1 INTRODUCTION

With the number of people living with HIV (PLHIV) accessing treatment increasing, the growing ageing population of PLHIV and with the move closer to epidemic control, implementation and scale‐up of innovative, efficient and simplified service delivery models to ensure lifelong antiretroviral therapy (ART) adherence and virologic suppression are essential to providing quality person‐centred care while also reducing strain on the healthcare system [1].

Historically, HIV service delivery was based on a one‐size fits all approach, where clients received undifferentiated services, which required multiple visits to a health facility for clinical consultations and to obtain ART [2]. Now, service delivery takes a person‐centred approach in which the diverse preferences, needs and expectations of clients are taken into consideration, allowing for reduced visits to a health facility, enhanced quality of care, improved treatment outcomes for clients and redirecting of resources to focus on those most at need of more intensive support [2, 3].

The Uganda Ministry of Health (MoH) first recommended differentiated service delivery (DSD) in their 2016 consolidated HIV prevention, care and treatment guidelines as a critical strategy to enable Uganda to achieve the UNAIDS 90‐90‐90 goals [4]. In addition to supporting efforts to rapidly expand the provision of ART to individuals, the Uganda MoH recommends six facility and community‐based differentiated antiretroviral therapy (DART) models to ensure that clients are empowered to manage their own care, reduce wait times at facilities, support continuity of treatment and linkage to supportive services [1]. The six DART models include: facility‐based individual management (FBIM), a more intensive model designed for clinically unstable and complex clients; facility‐based group (FBG) for unstable/complex or clinically stable clients; and four less‐intensive models for clinically stable clients, including facility‐based, fast‐track drug refill (FTDR), community client‐led ART delivery (CCLAD), community drug distribution point (CDDP) and community pharmacy [1].

Healthcare workers assess client eligibility criteria per Uganda MoH guidelines for DART models upon initial enrolment. Clients are clinically unstable if their viral load is unsuppressed, if they are newly enrolled receiving ART or if their viral load is unknown. Clients who do not yet meet the definition of clinically stable are placed into a more intensive DART model, such as FBIM or FBG, by their provider irrespective of their preferences to facilitate closer clinical monitoring. Clinically stable clients receive information about all available models and may opt for any DART model, including intensive and less‐intensive models. Client preference varies, and circumstances evolve thus requiring healthcare providers to adjust the model of service delivery. By routinely assessing client preferences for DART models, we can provide an evidence base of which models work for clients based on certain demographic factors and an understanding of the evolving needs of clients [5].

To evaluate whether a client receiving their preferred model is associated with clinical outcomes, we developed a DSD client preference, quality improvement tool to quantify the proportion of clients accessing their preferred DART model and compare the clinical outcomes of clients currently in their preferred DART models and those not in their preferred DART models.

## 2 METHODS

### 2.1 Study design and participants

A cross‐sectional study was conducted between January and February 2022 using the DSD client preference tool to understand client preferences for DART models and community linkages for support services. Our study included clients accessing care from the selected 113 referrals, general hospitals and health centres. Sample calculation estimated that 79% of all adult ART clients had been enrolled in one of the six DART models (FBG, FBIM, FTDR, CCLAD, CDDP or community pharmacy). With a normal standard deviation (1.96 and 95 CI), precision corresponding to a confidence interval of 5% and a design effect of three, a sample of 735 was needed for the study. To perform subgroup analyses, the sample was recomputed to factor DART models (FBG, FBIM, FTDR, CCLAD, CDDP and community pharmacy). Thus, the sample of 735 was multiplied by the seven sets required for disaggregated analysis to obtain 5145. Adjusting for a non‐response of 5% gives a sample of 5402. The sample was adjusted to cater for facility type (private not‐for‐profit [PNFP] and public health facilities), yielding a sample of 6137 clients. The study was approved by The AIDS Support Organization (TASO) Institutional Review Board under approval #TASOREC/030/2021‐UG‐REC‐009.

### 2.2 Study sites

The study was conducted in 113 sites in nine regions and included public and PNFP health facilities (sites). Study sites were selected to capture the variations in regional settings and levels of health facilities. All levels of health facilities were included, namely; regional referral hospital, general hospital, Health Center IV (HCIV) and Health Center III (HCIII), to include clients in all service delivery models, such as CCLAD and CDDP, who would otherwise not be found in regional referral hospitals or some general hospitals. Purposive sampling was used to select 10 health facilities from each region to reflect variation in model type, facility ownership and implementing partner (region). Thus, two general hospitals, four HCIV and four HCIII were selected from each region. No more than one health facility was selected per district except for the purpose of boosting the sample to reflect various geographical areas.

### 2.3 Sampling procedure

The sample frame included patients who received antiretroviral treatment in each selected facility. Sampling was purposive to include clients enrolled in different DSD models and age categories of 0–14, 15–24 and 25+ that would best answer the study questions. The selection and interviewing of clients were conducted over a period of 1 month when the desired sample was attainable across DSD models and age categories. We used cell‐based weighting on the predetermined sample size for each DSD group and age category so that results represent the clients in the selected sites. Relatedly, sites were purposively selected to include sites with all the DSD models attaining a participation rate of 97% of the desired clients.

### 2.4 The DSD client preference tool

**Figure 1:**
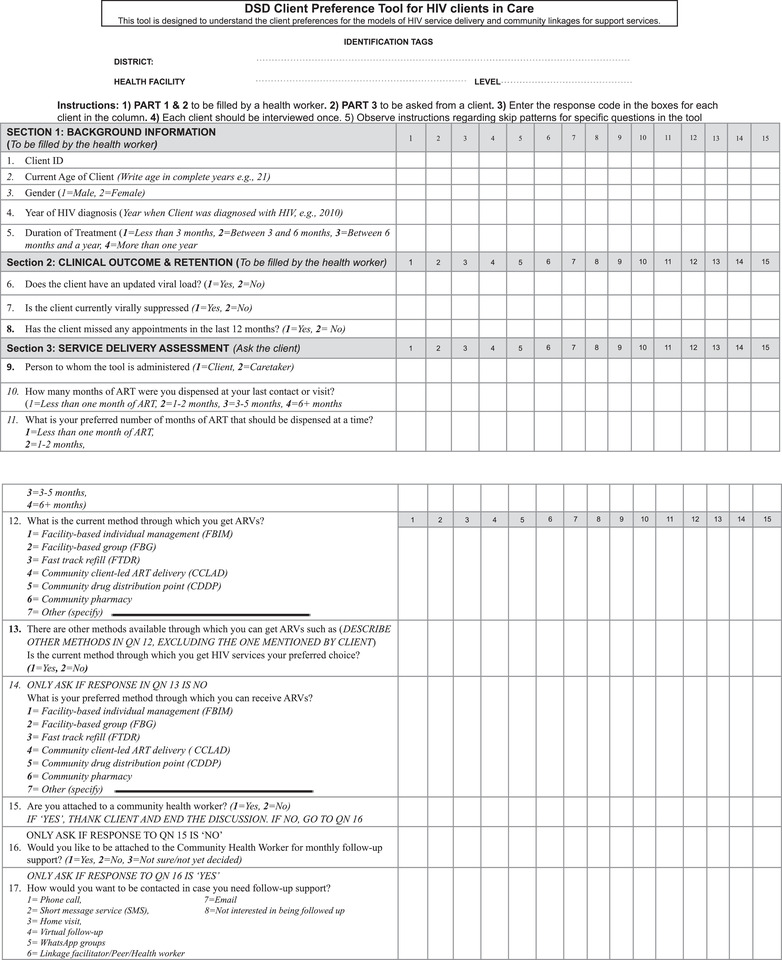
DSD client preference tool shows the client preference tool which contains 17 questions split into three sections, and it aims to compare client preferences for different DART models and their impact on HIV viral load suppression. The tool uses missed appointment data to gauge continuity of treatment, with clients who have not missed an appointment in the past year considered to have continuous treatment (see File S1 for copy of tool). Abbreviations: DSD, differentiated service delivery; DART, differentiated antiretroviral therapy.

The client preference tool (Figure 1) was drafted and discussed with subject matter experts through three iterative review meetings to generate the pre‐testing version. The tool was then pre‐tested, and the final tool was then adapted. The tool was composed of 17 questions divided into three sections: (1) background information; (2) clinical outcomes and retention; and (3) service delivery assessment. Sections 1 and 2 were abstracted from the client files by the health worker before or immediately after the interview. Section 1 contained information regarding client ID, current age, gender, year of HIV diagnosis and duration of treatment broken down by periods of 3 months. This information was used to compare what groups of people prefer certain DART models and the effect on HIV viral load suppression. The second section pertained to treatment outcomes of viral load tests, viral load suppression (<1000 copies/ml) and missed appointments; a proxy for continuity of treatment and defined as a client who did not miss an appointment in the last 12 months at the time of the study.

### 2.5 Dissemination of client preference tool

The tool, as well as standardized guidelines for data collection across the regions, was disseminated to USAID‐supported implementing partners through an orientation. One‐on‐one dissemination meetings with implementing partner data collection teams followed. The only inclusion or exclusion criteria for the clients to be interviewed using the DSD client preference tool was that the client was a current member of either a community or facility‐based DART model. Each client was only interviewed once.

### 2.6 Data collection

Data were collected from 113 referral and general hospitals and health centres purposely selected to capture all DSD client categories from 74 districts. Clients receiving ART accessing care from the sampled sites were eligible for inclusion. Selection of clients was done conveniently by interviewing clients within the period of data collection of 2 weeks irrespective of their appointment date until the desired number was attained.

Each participating health facility interviewed all their community pharmacy clients, a minimum of 16 CCLAD clients and 16 CDDP clients, 32 FBIM and 32 FTDR clients. The study set out to interview an equal number of men and women out of the sample allocated per facility per the DART model. However, in some cases, more women than men were interviewed when there were no more men in the model. If clients were infants, youth or adolescents, parents or guardians responded to the interview questions. Patient preference analysis was done during routine care by providers to align services to client preferences and, therefore, client consent was waived. When the total number of clients to be interviewed per model was not achieved in a selected health facility, the number was boosted by clients in the same DART model in another health facility. The tool was administered by a health worker or a community health worker to the eligible clients as part of routine care. All the completed data tools were collected for processing and analysis at a central place.

### 2.7 Data analysis

Descriptive statistics were used to analyse client demographic characteristics, clinical outcomes and clients’ preferences for care and presented as numbers and proportions. Sub‐population analyses were done to distinguish current DART models, client preferences and outcomes. Cross‐sectional comparisons of clinical outcomes and patient preferences were done to determine the interaction between client preferences and predefined treatment outcomes by comparing the outcomes of clients.

## 3 RESULTS

### 3.1 Characteristics of clients

The overall study cohort included 6376 clients ages 1–92 years (Table 1). The clients were selected from 113 facilities across nine regions, with the Mid‐Northern and Southwestern regions accounting for 1656 (26%) and 1363 (21%) of the sample, respectively. Table 1 illustrates the demographic distribution of clients by age (<15 and 15+), gender, region of residence and current Antiretroviral (ARV) mode. This table also highlights the frequency of missed appointments, community health worker presence and viral load suppression as binary outcomes. Age distribution varied widely, with adults (15+) accounting for almost 84% of the sample population and children (<15) accounting for 16%. More than half (58%) of the clients in the sample identified as female. Two thousand and seven (32%) of the clients had a reported missed appointment in the last 12 months and over 5113 (80%) of clients were virally suppressed. While most clients reported that they were in their preferred ARV model, 1573 (25%) reported that they were not in their preferred method.

**Table 1:** Baseline cohort demographics.

	Overall (*N* = 6376)
**Age**	
Mean (SD)	32.9 (16.3)
Median [Min, Max]	33.0 [1.00, 92.0]
Missing	4 (0.1%)
**Age category**	
<15	1022 (16.0%)
15+	5350 (83.9%)
Missing	4 (0.1%)
**Sex**	
Female	3741 (58.7%)
Male	2565 (40.2%)
Missing	70 (1.1%)
**Region**	
Central	1 (0.0%)
East Central	1062 (16.7%)
Karamoja	590 (9.3%)
Mid‐Eastern	635 (10.0%)
Mid‐Northern	1656 (26.0%)
Mid‐Western	1 (0.0%)
South Western	1363 (21.4%)
UPMB/LSDA^*^	984 (15.4%)
West Nile	84 (1.3%)
**Missed appointments in last 12 months**	
No missed appointment	4280 (67.1%)
Missed appointment	2007 (31.5%)
Missing	89 (1.4%)
**Current ARV mode**	
Community client‐led ART delivery (CCLAD)	781 (12.2%)
Community drug distribution point (CDDP)	809 (12.7%)
Community pharmacy	184 (2.9%)
Facility‐based group (FBG)	776 (12.2%)
Facility‐based individual management (FBIM)	2018 (31.7%)
Fast‐track drug refill (FTDR)	1808 (28.4%)
**ARV mode preference**	
No	1573 (24.7%)
Yes	4803 (75.3%)
**Viral load suppression**	
No	1071 (16.8%)
Yes	5113 (80.2%)
Missing	192 (3.0%)

*UPMB/LSDA refers to Uganda Protestant Medical Bureau Local Service Delivery for Health and HIV/AIDS Activity, a partner organization that was analysed as a region because they work with all private not‐for‐profit institutions.

### 3.2 Client DART preferences and clinical outcomes

**Figure 2:**
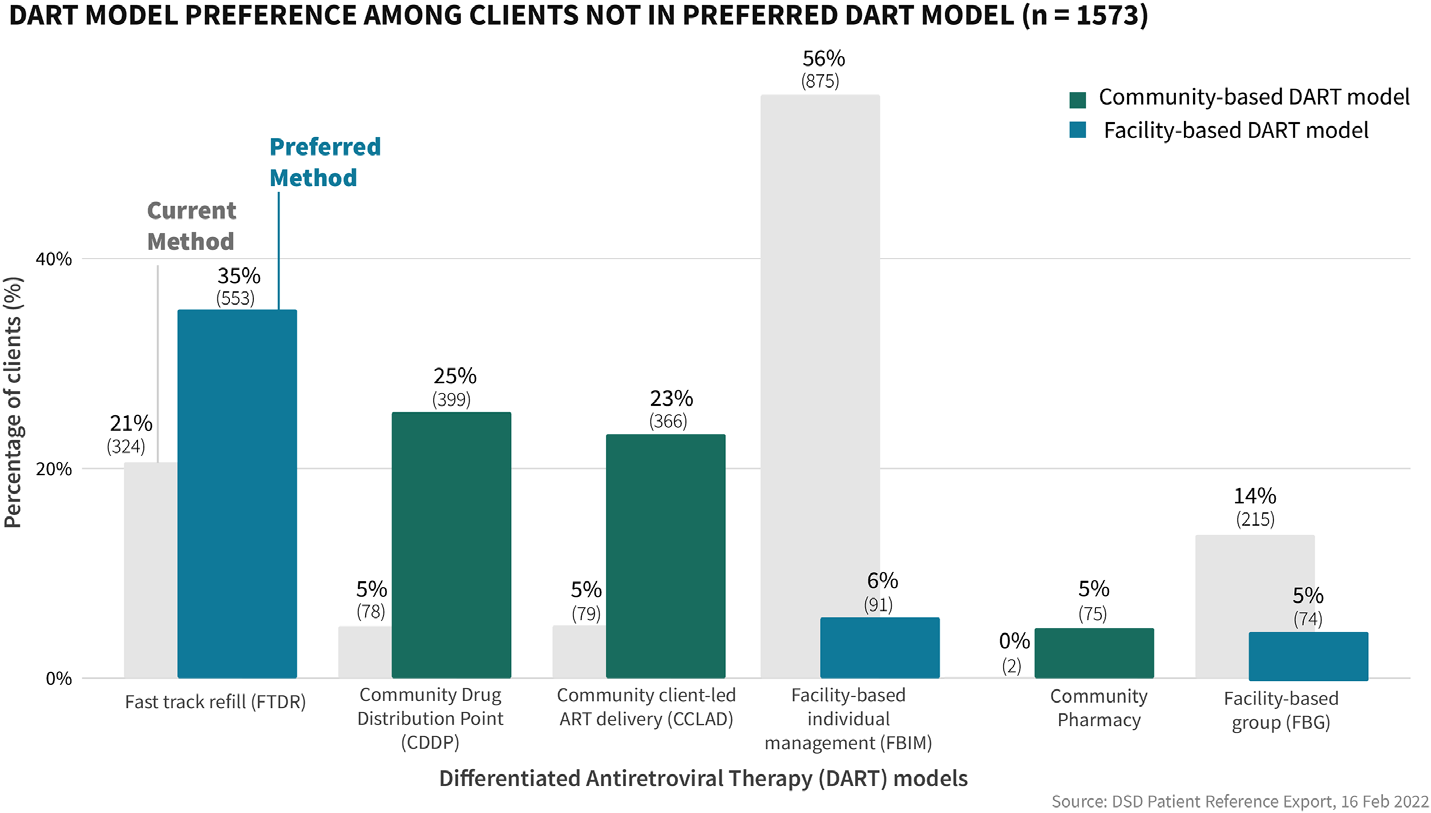
Differentiated antiretroviral therapy (DART) model preference among clients not in preferred DART model illustrates the distribution of clients’ preferred DART model among clients who were not currently enrolled in their preferred model.

**Figure 3:**
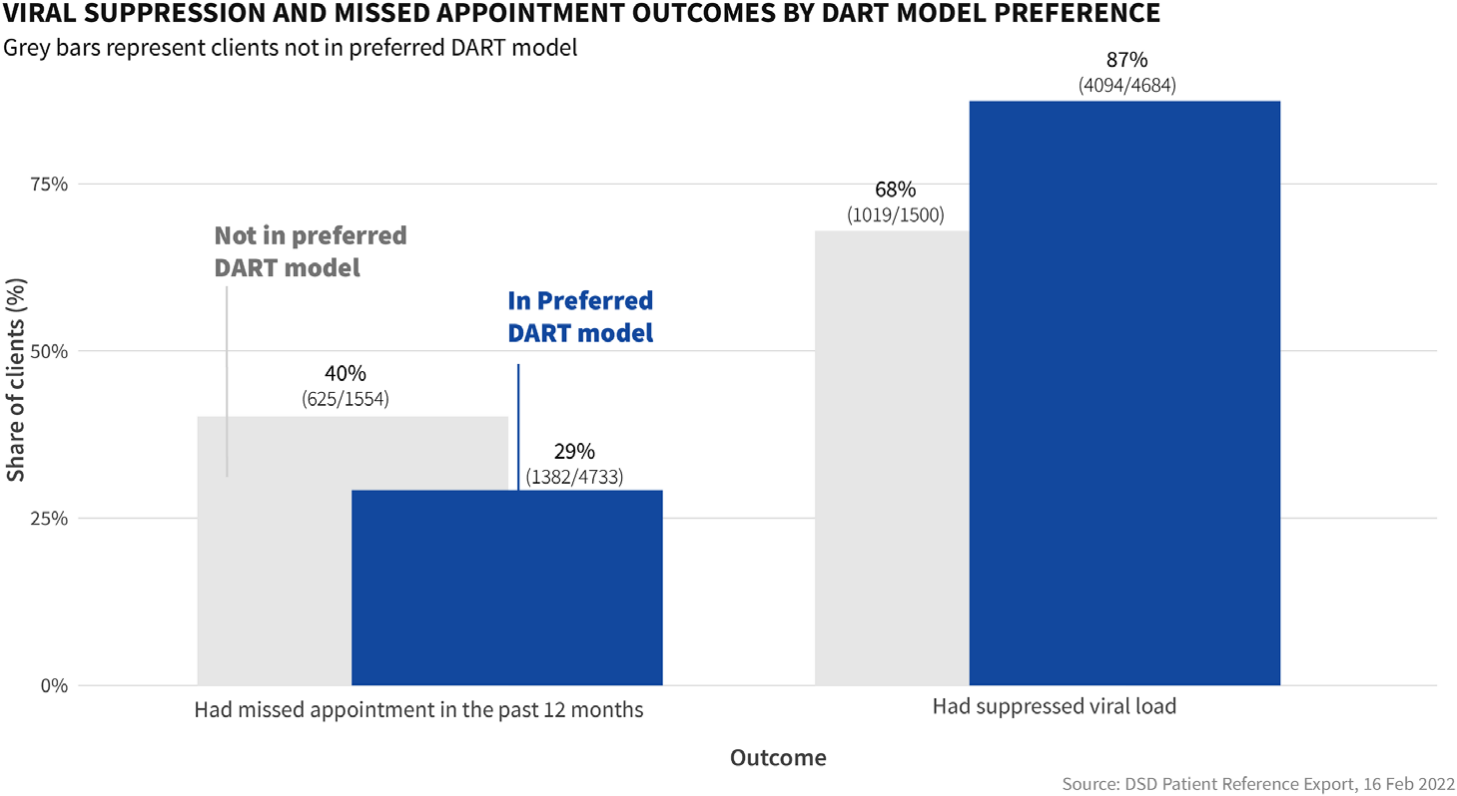
Viral suppression and missed appointment outcomes by Differentiated antiretroviral therapy (DART) model preference compares the viral load suppression rates and missed appointments outcomes between clients accessing their preferred DART model and those not accessing their preferred model.

Of the 25% (1573) of clients in the sample who were not accessing their preferred DART model, 91% were currently receiving facility‐based DART and 875 (56%) were currently accessing FBIM (Figure 2). Most clients not accessing their preferred model preferred community‐based DART models 840 (53%), 553 (35%) of clients in this group preferred the fast‐track refill model. Viral load suppression was higher among clients accessing their preferred DART model at 87% (4043/4623) (Figure 3) compared to clients not accessing their preferred DART model at 68% (1007/1482) (Table 2). The rate of missed appointments was lower at 69% (133) for clients accessing their preferred DART models compared to 31% (601) (Table 3) among clients not enrolled in the DART model of their choice. Over half of the cohort was enrolled in FBIM and FTDR methods, at 1872 (31%) clients and 1765 (29%) clients, respectively.

**Table 2:** Viral suppression outcomes by demographic and clinical characteristics.

	No (*n* = 1055)	Yes (*n* = 5050)	Overall (*N* = 6105)
**Age category**			
<15	222 (21.0%)	760 (15.0%)	982 (16.1%)
15+	833 (79.0%)	4286 (84.9%)	5119 (83.8%)
Missing	0 (0%)	4 (0.1%)	4 (0.1%)
**Sex**			
Female	574 (54.4%)	3005 (59.5%)	3579 (58.6%)
Male	467 (44.3%)	1993 (39.5%)	2460 (40.3%)
Missing	14 (1.3%)	52 (1.0%)	66 (1.1%)
**ARV mode preference**			
No	475 (45.0%)	1007 (19.9%)	1482 (24.3%)
Yes	580 (55.0%)	4043 (80.1%)	4623 (75.7%)
**Current ARV mode**			
Community client‐led ART delivery (CCLAD)	28 (2.7%)	728 (14.4%)	756 (12.4%)
Community drug distribution point (CDDP)	48 (4.5%)	743 (14.7%)	791 (13.0%)
Facility‐based group (FBG)	92 (8.7%)	647 (12.8%)	739 (12.1%)
Facility‐based individual management (FBIM)	809 (76.7%)	1063 (21.0%)	1872 (30.7%)
Fast‐track refill (FTDR)	78 (7.4%)	1687 (33.4%)	1765 (28.9%)
Community pharmacy	0 (0%)	182 (3.6%)	182 (3.0%)

**Table 3:** Missed appointment outcomes (in the last 12 months) by demographic and clinical characteristics.

	No missed appointment (*n* = 4169)	Missed appointment (*n* = 1936)	Overall (*N* = 6105)
**Age category**			
<15	591 (14.2%)	391 (20.2%)	982 (16.1%)
15+	3576 (85.8%)	1543 (79.7%)	5119 (83.8%)
Missing	2 (0.0%)	2 (0.1%)	4 (0.1%)
**Sex**			
Female	2438 (58.5%)	1141 (58.9%)	3579 (58.6%)
Male	1679 (40.3%)	781 (40.3%)	2460 (40.3%)
Missing	52 (1.2%)	14 (0.7%)	66 (1.1%)
**ARV mode preference**			
No	881 (21.1%)	601 (31.0%)	1482 (24.3%)
Yes	3288 (78.9%)	1335 (69.0%)	4623 (75.7%)
**Current ARV mode**			
Community client‐led ART delivery (CCLAD)	613 (14.7%)	143 (7.4%)	756 (12.4%)
Community drug distribution point (CDDP)	624 (15.0%)	167 (8.6%)	791 (13.0%)
Community pharmacy	126 (3.0%)	56 (2.9%)	182 (3.0%)
Facility‐based group (FBG)	458 (11.0%)	281 (14.5%)	739 (12.1%)
Facility‐based individual management (FBIM)	1119 (26.8%)	753 (38.9%)	1872 (30.7%)
Fast‐track refill (FTDR)	1229 (29.5%)	536 (27.7%)	1765 (28.9%)

## 4 DISCUSSION

Despite the large‐scale rollout of DART models in various formats across multiple countries, there is a shortage of evidence to document the purported benefits of the new models in routine implementation or clinical outcomes [6]. Our study, therefore, explored client DART preference in relation to clinical outcomes, based on the hypothesis that client care needs change throughout the life course, clients bring an understanding of their own needs, client participation in decision‐making can facilitate greater engagement and HIV outcomes are better when clients are in their preferred DART model. We found better clinical outcomes among clients accessing their preferred DART models. This finding could be explained by the fact that differentiated models of service delivery are tailored to addressing retention and access needs, generate greater client satisfaction, lower cost to both providers and clients, and create efficient and convenient service delivery [7, 8]. A major strength of this study is that it was done in a routine healthcare setting and covered sub‐population types from FBIM, FBG, FTDR, CCLAD and community pharmacy [4]. The results are, therefore, generalizable as far as they represent perspectives across the recommended DART models in Uganda.

Studies have found that DART models substantially reduce costs to clients, primarily for transport and time [9]. We, therefore, suggest both from our study and from other jurisdictions, a need to scale‐up the dispensing of 6 or more months of drugs for clients to reduce the frequency of medication refill visits, especially in resource‐constrained settings, such as in Uganda, especially if preferred by the clients.

The World Health Organization recommends monitoring ART efficacy using VL testing performed at 6 months following initiation, and annually afterwards [10]. In Uganda, all clients living with HIV should receive a viral load test 6 months after initiating treatment and annually thereafter; for adult clients who are suppressed and 6 monthly for non‐suppressed clients and children and adolescents. Our study determined whether clients eligible for viral load had received a repeated viral load test. We discovered that clients accessing their preferred DART model mostly had an up‐to‐date viral load. DART‐related interventions to support VL monitoring could explain in part the observed results and help address barriers to VL testing, especially among children. Enrolling clients to access a DART model of their preference would have complementary benefits of overcoming constraints associated with VL testing, such as long distances and costs associated with travelling to the health facilities. Other studies have documented a myriad of barriers to HIV care from both provider, client and health system perspectives [6], such as frequent clinic visits for clinical evaluations and drug refills, long waiting times in ART clinics, long distances and costs to travel to the health facilities [11].

One‐quarter of clients are not accessing their preferred model, which presupposes a proportion of clients remaining in a more intensive model of care, whose outcomes may be worse. The gap in enrolment according to client choice is partly explained by the reservations to enrol unstable clients to access other models other than FBIM. Clients not accessing their preferred model could miss one or more benefits, such as higher adherence to ART and continuity on treatment, reduced per‐client cost of providing ART and decreased waiting time. These among other benefits have been well‐postulated in other studies on differentiated models of HIV treatment [12].

### 4.1 Limitations of the study

Our study had some limitations, which are common with client preference studies. The study was cross‐sectional and could not explore how long clients were in their current DART model at the time of the survey, changes in a client's DART preferences and clinical outcomes over time. Stemming from this limitation, future studies should consider longer‐term follow‐up as it is critical to observe any changes to clinical outcomes due to evolving client preferences. Secondly, there is some selection bias in our participant inclusion criteria because we only included clients attending a healthcare visit at the facility, thus excluding many clients in the community and those who had missed their appointment or fallen out of care and we extended participation to clients enrolled in FBIM, a DART model for clinically unstable/complex clients needing intensive support. The highest proportion of clients not in the model of their choice were enrolled in FBIM; and because FBIM includes clients who are largely virologically non‐suppressed, unlike the other models, this may skew viral load suppression results by group. However, when we exclude the FBIM patients, viral load suppression was still higher among clients accessing their preferred DART model (95%) compared to clients not accessing their preferred DART model (90%). Additionally, to address the limitation on including clients in the community who missed appointments and could not have their preference assessed, we analysed all clients who attended the clinic during the project period, whether or not they were on appointment. Table 1 illustrates that 31.5% of the clients included in the analysis had missed an appointment in the 12 months prior to the data collection.

Although the study included clients who attended the clinic during the study period, regardless of whether or not they had a scheduled appointment, the convenience sampling methodology used in this study limits its ability to generalize the findings to the broader population of PLHIV. As a cross‐sectional study, the design did not allow for causal inference, and the results should be interpreted with caution.

Nevertheless, the descriptive analysis provided valuable insights into the prevalence of missed appointments and their association with client preferences for appointment scheduling. To further explore the relationship between client preferences and clinical outcomes, future research using more robust study designs, such as randomized controlled trials or longitudinal studies, would be needed.

## 5 CONCLUSIONS

One‐fourth of clients’ current DART model were not enrolled in their preferred choice, and clients enrolled in their preferred choice had higher rates of viral load suppression and fewer missed appointments. Some of these observed findings may be driven by fewer stable clients being required to remain in more intensive models until clinical milestones are met; however, it may also reflect a slowness to move eligible clients back into a preferred model. While clients in their preferred model of service delivery had timely viral load monitoring, these clients are also mostly enrolled in less‐intensive models with fewer clinical touchpoints. Knowing that different DART models bring different benefits (e.g. social support, anonymity and extra engagement with HCWs), and that clients likely have the best understanding of their changing individual needs, clients should be actively engaged in deciding how they receive care. Continuous assessment of client preferences for DART models using a quality improvement tool, counselling and assignment of clients to models of choice is essential in improving client experience of care and ultimately clinical outcomes. Additionally, a longitudinal study of client preferences is essential to better understand the impact of client preferences for DART models on clinical outcomes.

## COMPETING INTERESTS

The authors declare that they have no competing interests.

## AUTHOR CONTRIBUTION

Conceived, designed the analysis and collected the data: Esther K. Karamagi Nkolo, Simon Sensalire, Juliana Nabwire Ssali, Immaculate Ddumba, Jacqueline Calnan Contributed to the data & performed the analysis: Nelly Maina, and Karishma Srikanth Wrote the paper: Esther K. Karamagi Nkolo, Jessica Clinkscales Ejike, Simon Sensalire, Juliana Nabwire Ssali, Immaculate Ddumba, Jacqueline Calnan,Carolina Gonzalez, Nelly Maina, Melaku Dessie, Lauren Bailey, Ugochukwu Amanyeiwe,Thomas Minior, Karishma Srikanth, Herbert Kadama ,Khushi Patel, Dina Patel

## FUNDING

This article was made possible by the support of the American people through the United States Agency for International Development (USAID) under the U.S. President's Emergency Plan for AIDS Relief (PEPFAR).

## DISCLAIMER

The views in this abstract are those of the authors and do not necessarily represent the views of the U.S. Agency for International Development, University Research Corporation LLC or the Uganda Ministry of Health.

## Supporting information


**Supporting Information S1**: DSD client preference tool. Tool used to assess client preference for DART models.

## Data Availability

The data that support the findings of this study are available from the corresponding author upon reasonable request.
