# Supplementary material for: Clients in Uganda accessing preferred differentiated antiretroviral therapy models achieve higher viral suppression and are less likely to miss appointments: a cross‐sectional analysis
Source: J Int AIDS Soc. 2023 Jul 6;26(Suppl 1):e26122. doi: 10.1002/jia2.26122 (PMC10323312; doi:10.1002/jia2.26122)
Supplement: Supplementary file 1 — Supporting Information S1: DSD client preference tool. Tool used to assess client preference for DART models. [file JIA2-26-e26122-s001.pdf]

DSD Client Preference Tool for HIV clients in Care

This tool is designed to understand the client preferences for the models of HIV service delivery and community linkages for support services.

IDENTIFICATION TAGS

DISTRICT: ....

HEALTH FACILITY .... **LEVEL**.......................................

Instructions: 1) PART 1 & 2 to be filled by a health worker**. 2) PART 3** to be asked from a client**. 3)** Enter the response code in the boxes for each client in the column**. 4)** Each client should be interviewed once. 5) Observe instructions regarding skip patterns for specific questions in the tool

**SECTION 1: BACKGROUND INFORMATION** **(***To be filled by the health worker***)**	1	2	3	4	5	6	7	8	9	10	11	12	13	14	15
1. Client ID															
2. Current Age of Client *(Write age in complete years e.g., 21)*															
3. Gender (*1=Male, 2=Female)*															
4. Year of HIV diagnosis (*Year when Client was diagnosed with HIV, e.g., 2010)*															
5. Duration of Treatment (***1****=Less than 3 months,* ***2****=Between 3 and 6 months,* ***3****=Between 6 months and a year,* ***4****=More than one year*															
**Section 2: CLINICAL OUTCOME & RETENTION (***To be filled by the health worker)*	1	2	3	4	5	6	7	8	9	10	11	12	13	14	15
6. Does the client have an updated viral load? (***1****=Yes,* ***2****=No)*															
7. Is the client currently virally suppressed *(**1**=Yes, **2**=No)*															
**8.** Has the client missed any appointments in the last 12 months? *(**1**=Yes, **2**= No)*															
**Section 3: SERVICE DELIVERY ASSESSMENT** *(Ask the client)*	1	2	3	4	5	6	7	8	9	10	11	12	13	14	15
**9.** Person to whom the tool is administered *(**1**=Client, **2**=Caretaker)*															
10. How many months of ART were you dispensed at your last contact or visit? (*1=Less than one month of ART,* ***2****=1-2 months,* ***3****=3-5 months,* ***4****=6+ months*															
11. What is your preferred number of months of ART that should be dispensed at a time? ***1****=Less than one month of ART,* ***2****=1-2 months,*															

3*=3-5 months,*<br>***4****=6+ months)*															
	1	2	3	4	5	6	7	8	9	10	11	12	13	14	15
12. What is the current method through which you get ARVs?<br>***1****= Facility-based individual management (FBIM)*<br>***2****= Facility-based group (FBG)*<br>***3****= Fast track refill (FTDR)*<br>***4****= Community client-led ART delivery (CCLAD)*<br>***5****= Community drug distribution point (CDDP)*<br>***6****= Community pharmacy*<br>*7= Other (specify)* ______________________															
**13.** There are other methods available through which you can get ARVs such as (*DESCRIBE OTHER METHODS IN QN 12, EXCLUDING THE ONE MENTIONED BY CLIENT*)<br>Is the current method through which you get HIV services your preferred choice?<br>***(1****=Yes****, 2****=No****)***															
14. *ONLY ASK IF RESPONSE IN QN 13 IS NO*<br>What is your preferred method through which you can receive ARVs?<br>***1****= Facility-based individual management (FBIM)*<br>***2****= Facility-based group (FBG)*<br>***3****= Fast track refill (FTDR)*<br>***4****= Community client-led ART delivery (CCLAD)*<br>***5****= Community drug distribution point (CDDP)*<br>***6****= Community pharmacy*<br>*7= Other (specify)* ______________________															
15. Are you attached to a community health worker? *(**1**=Yes, **2**=No)*<br>*IF 'YES', THANK CLIENT AND END THE DISCUSSION. IF NO, GO TO QN 16*															
ONLY ASK IF RESPONSE TO QN 15 IS 'NO'<br>16. Would you like to be attached to the Community Health Worker for monthly follow-up support? *(**1**=Yes, **2**=No, **3**=Not sure/not yet decided)*															
ONLY ASK IF RESPONSE TO QN 16 IS 'YES'<br>17. How would you want to be contacted in case you need follow-up support?<br>*1= Phone call,* *7=Email*<br>*2= Short message service (SMS),* *8=Not interested in being followed up*<br>*3= Home visit,*<br>*4= Virtual follow-up*<br>*5= WhatsApp groups*<br>*6= Linkage facilitator/Peer/Health worker*															
